# AI-driven multimodal imaging: unveiling hidden cardiac vulnerabilities in osteogenesis imperfecta

**DOI:** 10.1097/MS9.0000000000004737

**Published:** 2026-01-26

**Authors:** Rania Ahmad, Hameed Ullah, Samia Ahmad, Muhammad Talha, Mahnoor Fatima

**Affiliations:** a Department of Medicine, Quaid e Azam Medical College, Bahawalpur, Pakistan; b Department of Medicine, CMH Institute of Medical Sciences, Bahawalpur, Pakistan; c Department of Medicine, King Edward Medical University, Lahore, Pakistan; d Department of Medicine, Shaheed Ziaur Rahman Medical College, Rajshahi Medical University, Bogura Bangladesh

*To the Editor,*

Osteogenesis imperfecta (OI), also referred to as brittle bone disease, is a rare heterogeneous skeletal abnormality with an estimated frequency of 1 in 15 000–20 000 live births. Clinically, it is marked by growth deficiency, bone anomalies, repeated fractures, and bone fragility. The majority of cases are caused by autosomal dominant mutations in the type I collagen-encoding genes COL1A1 and COL1A2. Depending on the severity, it can be classified as type I, II, III, or IV. The hypomorphic (milder) version of OI is type I^[1]^. Since collagen type I provides cardiac tissue with its tensile strength and flexibility, when it is defective in OI type I patients, it results in heart valve dysfunction, heart failure, atrial fibrillation, hypertension, and dilated aortic root^[2]^. The promise of artificial intelligence (AI)-based imaging, including computed tomography (CT), magnetic resonance imaging (MRI), and echocardiography, for improved cardiac screening in hypomorphic OI is highlighted in this letter. This is in line with the TITAN Guidelines on the need for transparency in AI use in healthcare^[3]^.

AI-driven multimodal imaging is an advanced system that provides a significant breakthrough in cardiac diagnoses by combining deep-learning algorithms with CT, cardiac magnetic resonance (CMR), and echocardiography. AI increases picture quality, automates segmentation, and detects cardiac and valvular abnormalities, including ischemia and fibrosis, using convolutional and recurrent neural networks. These methods allow for quantitative and reproducible assessments of heart morphology and function. Extending these capabilities to genetically induced disorders like OI is clinically relevant, as a recent analysis found an increased frequency of aortic root dilatation, valve dysfunction, and subclinical myocardial impairment in OI^[2,4]^. Integrating AI-enhanced CT, CMR, and echocardiographic data could result in a precision-based framework for early diagnosis, longitudinal monitoring, and risk assessment of cardiac involvement in OI.

Recent advances in AI have enabled the integration of multimodal imaging techniques for cardiovascular assessment, providing a new and improved platform to evaluate cardiac involvement in rare connective tissue disorders such as OI. According to a recent study, multimodal AI systems can combine information from various imaging methods, including echocardiography, CT, and cardiac MRI, to enhance diagnostic accuracy, automate cardiac function evaluations, and improve the prediction of disease outcomes^[5]^. Additionally, a study shows the growing usefulness of AI in cardiac imaging workflows, emphasizing its ability to speed up image interpretation, reduce inter-observer variability, and strengthen risk assessment^[6]^. Collectively, these studies support the case for implementing AI-driven multimodal imaging techniques to monitor cardiac health in OI patients.

The effectiveness of AI in cardiovascular imaging depends heavily on standardized, high-quality training data. However, there are significant obstacles due to differences in imaging procedures, equipment calibration, and patient demographics throughout institutions. There are practical and legal obstacles to building databases across institutions. Concerns are also raised by ethical and legal issues, such as accountability for AI-related mistakes and intrinsic data bias. Insufficient diversity in datasets can intensify sociocultural bias, resulting in disparate outcomes and jeopardizing patient safety and clinical trust^[6,7]^.

To increase the accuracy of cardiac involvement diagnosis associated with OI, future research should concentrate on creating larger, multi-centric AI-driven multimodal imaging databases. Early detection of small myocardial alterations can be improved by integrating cardiac MRI, CT, and echocardiography using advanced deep learning. Transparent algorithms, ethical supervision, and clinician-scientist cooperation are necessary to ensure accuracy in this uncommon genetic disorder and ensure dependable, patient-centered application.

## Data Availability

Not applicable to this study.
